# Involuntary retirement and organizational climates: a multilevel study of older workers

**DOI:** 10.1093/geronb/gbag128

**Published:** 2026-07-01

**Authors:** Camilla Marabini, Kène Henkens, Hanna van Solinge

**Affiliations:** Demographic Change and the Labour Market, Netherlands Interdisciplinary Demographic Institute, The Hague, The Netherlands; Department of Health Sciences, University Medical Center Groningen, Groningen, The Netherlandsz; Demographic Change and the Labour Market, Netherlands Interdisciplinary Demographic Institute, The Hague, The Netherlands; Department of Health Sciences, University Medical Center Groningen, Groningen, The Netherlandsz; Demographic Change and the Labour Market, Netherlands Interdisciplinary Demographic Institute, The Hague, The Netherlands; Department of Health Sciences, University Medical Center Groningen, Groningen, The Netherlandsz; (Social Sciences Section)

**Keywords:** Age-friendly environments, Employment, Work-related issues, Life course agency, Multilevel or hierarchical model

## Abstract

**Objectives:**

Maintaining control over the retirement transition is crucial for post-retirement well-being. Yet, studies show that between 10% and 40% of employees experience involuntary retirement. This study investigates 3 separate reasons for involuntary retirement: poor health, organizational pressures, and reaching the quasi-mandatory pension age. Guided by a life-course framework of agency and structure, we analyze how collective and individual perceptions of development, accommodation, and post-retirement work climates shape separate reasons for involuntary retirement, as opposed to voluntary retirement.

**Methods:**

Using multilevel panel data from the Netherlands, we estimate multilevel logistic regression models on 3,735 respondents nested in 501 organizations. We assess how organizational climates, which vary systematically between organizations, and individual-relative perceptions, which vary systematically within organizations, are associated with different involuntary retirement reasons.

**Results:**

Reaching the quasi-mandatory retirement age was the main reason for involuntary retirement. Across organizations, a supportive organizational climate for development reduced involuntary retirement. At the individual level, employees who perceived more supportive accommodation and post-retirement work climates relative to their colleagues were less likely to experience involuntary retirement. Organizational and individual-relative climates revealed distinct associations with different reasons for involuntary retirement.

**Discussion:**

Our results suggest that involuntary retirement is a multidimensional phenomenon shaped by micro-, meso-, and macro-level constraints. Supportive organizational policies and practices enhance employees’ agency, with some differences depending on the reason for involuntary retirement. Policies encouraging training of older workers may increase both labor force participation and older employees’ choices as they near retirement.

As the population in Western countries is rapidly aging, retaining older workers in the labor force is critical to sustaining economic productivity and reducing pressure on pension systems (OECD, 2025a, p. 6). Still, studies consistently show that between 10% and 40% of employees retire without intending to do so (Denton et al., 2013; Ebbinghaus & Radl, 2015; Szinovacz & Davey, 2005; Van Solinge & Henkens, 2007; Welsh et al., 2018). Involuntary retirement has consequences not only for society, as it constrains older workers’ labor force participation, but also for individual well-being. Retiring involuntarily has been associated with poorer physical and mental health after the retirement transition (Rhee et al., 2016), decreased short-term and long-term well-being (Radó & Boissonneault, 2020), and adjustment problems (Dingemans & Henkens, 2014). Involuntary retirement is often treated as an individual circumstance, yet it is embedded in organizational and institutional contexts that have received limited empirical attention so far (Stiemke & Hess, 2022). This raises two key questions: to what extent do organizational policies and practices shape involuntary retirement transitions? And how do they differently affect involuntary retirement due to poor health, organizational pressures, and mandatory retirement regulations?

Involuntary retirement has been defined as “the individual’s perception of the degree to which he or she retired voluntarily” (Beehr, 1986, p. 34), reflecting older workers’ perceived agency in the retirement transition (Szinovacz & Davey, 2005). Research typically treats involuntary retirement as a unidimensional construct. Many studies rely on a dichotomous distinction between voluntary versus involuntary transitions (Denton et al., 2013; Dorn & Sousa-Poza, 2010; Hyde & Dingemans, 2017; Radl, 2013; Szinovacz & Davey, 2005). However, older workers’ agency can be constrained in multiple ways. According to a multi-level retirement framework, such constraints may occur at the individual, organizational, and institutional levels (Moen, 2013; Szinovacz, 2012).

Poor health may limit an individual’s ability to continue to work (Denton et al., 2013), while redundancy or lay-offs result from employers’ decisions (Clarke, 2007). In addition, mandatory retirement regulations—still in place in many Western countries (OECD, 2025b, p. 36)—may restrict older workers’ agency by requiring them to exit employment at the state pension age (Fritz, 2024). Taken together, these reasons represent distinct dimensions, each with different antecedents, consequences, and policy implications. In this article, we therefore present a multi-dimensional measure of involuntary retirement, distinguishing between involuntary retirement because of poor health, organizational pressure, and mandatory retirement regulations, relative to voluntary retirement.

Research on involuntary retirement has predominantly focused on individual determinants. Existing findings suggest that personal resources such as better health, financial security, and social support are associated with greater agency in the retirement transition (Damman & Henkens, 2017; Stiemke & Hess, 2022). In contrast, considerably less attention has been paid to the characteristics of older workers’ organizational environments (Caines et al., 2025). At the organization level, employees in larger organizations appear less likely to retire voluntarily (Hofäcker et al., 2016; Radl, 2013). At the country level, factors such as rising unemployment and restrictive labor market regulations are associated with higher rates of involuntary retirement, likely mediated by employers’ decisions (Dorn & Sousa-Poza, 2010; Hyde & Dingemans, 2017).

While these studies highlight the importance of structural conditions shaping retirement transitions, far less attention has been paid to how older workers perceive their organizations’ policies and practices. Employers use human resource management toward older workers strategically within existing sectoral and national regulations (Lössbroek et al., 2019; Turek et al., 2020; Van Dalen et al., 2015). As such, they are likely to shape older workers’ perceived agency by offering—or restricting—access to organizational policies and practices that facilitate control over the retirement transition (Henkens & Marabini, 2026; Moen, 2013; Szinovacz, 2012).

Using a longitudinal, multilevel dataset with employees nested in work organizations, we assess how both collective and individual perceptions of organizational climates shape involuntary retirement. Organizational climates are defined as “the shared perceptions of and the meaning attached to the policies, practices, and procedures employees experience” (Schneider et al., 2013, p. 362). Our analysis distinguishes between organizational climates, as organization-level constructs, and individual climate perceptions, measured as deviation from the organizational mean. We examine how different organizational climates may operate across involuntary retirement processes.

This article contributes to the literature in three ways. First, we introduce a multi-dimensional indicator of involuntary retirement that captures perceived constraints at the individual (health), organizational (organizational pressures), and institutional (mandatory retirement) levels. Second, we advance research on retirement transitions by explicitly examining how organizational climates shape involuntary retirement, while accounting for established individual-level determinants. Third, we advance the field empirically by drawing on high-quality longitudinal, multilevel data. The NIDI Pension Panel Survey (Henkens et al., 2017) provides longitudinal observations of 3,735 respondents transitioning into retirement between 2015 and 2023, nested in 501 organizations.

This investigation is carried out in the Netherlands. In the Dutch pension system, the state pension age was 65 until 2013 and has since been gradually increased to 67 years in 2024. For most wage-employed workers, mandatory retirement at the state pension age is regulated in collective labor agreements and is generally enforced when reaching the state pension age (OECD, 2025b, p. 38). Continuing to work after state pension age is possible by entering a new employment contract if both the employer and the employee agree. We therefore also refer to this age as “quasi-mandatory retirement age.” Labor market participation of older workers in the Netherlands has been increasing, also due to reforms that blocked early exit routes. Moreover, Dutch employers are increasingly adopting age management practices such as training for older workers and accommodation practices in their work organizations (Turek et al., 2020).

## Theory

### Involuntary retirement as constrained agency

The life-course perspective posits that the life course is “actively created by individuals (…), within the confines of the social worlds in which they exist” (Settersten, 2003, p. 30). This idea is commonly summarized as the interplay between agency and structure. Individuals strive to shape their life course by maintaining control over important transitions (Heckhausen & Schulz, 1995). Retirement is a major later-life transition in which the interplay between agency and structure is evident. It is often argued that employees retire once the perceived benefits of retirement outweigh its costs (Feldman & Beehr, 2011). However, such considerations only come into play when a real choice exists (Szinovacz & Davey, 2005). Perceptions of retirement voluntariness can be seen as an indicator of late-life agency, reflecting the perceived degree of influence on the timing and nature of labor market exit (Damman & Henkens, 2017; Szinovacz & Davey, 2005).

From a life-course perspective, an individual’s perceived agency in retirement depends on structural conditions that may constrain choice (Moen, 1996). Employees experience retirement within work organizations, where formal policies and informal arrangements may facilitate or constrain their agency. In the following paragraphs, we first formulate hypotheses about how organizational and individual climate perceptions, reflecting employer policies and practices, relate to involuntary retirement. We then formulate hypotheses about how organizational climates differently affect involuntary retirement occurring for three reasons: poor health, organizational pressures, and reaching the quasi-mandatory retirement age.

### Organizational climates and involuntary retirement

Organizations vary significantly in the policies and practices that they offer to their employees. Their approach to an older workforce can influence retirement transitions. Some primarily adopt practices that keep older workers engaged in paid work, while others facilitate their gradual withdrawal from the workforce (Lössbroek et al., 2019). Van Dalen et al. (2015) offered a taxonomy of organizational practices for older workers, classifying them into development, accommodation, and exit. Development refers to training and job mobility; accommodation refers to ergonomic adaptations, reductions in hours and workload, and exemption from irregular or shift work; and exit refers to early retirement opportunities.

Organizational climates provide insights into how employees experience their work environment (Schneider et al., 2013). Previous research shows that organizational climates are strongly related to available HR policies and practices (Boehm et al., 2014). Most importantly, they signal perceived management support for using them. If organizations are not supportive of policies that are available “on paper,” older employees may not know of their existence or feel afraid to use them due to the potential consequences. For example, they may hesitate to disclose health conditions for fear of job loss, preventing them from obtaining necessary accommodations (Gignac et al., 2024).

While organizational climates represent shared perceptions, individuals within organizations may perceive climates more or less positively than colleagues (Joyce & Slocum, 1982). These individual differences in perception, which we refer to as “relative perceptions,” may arise from social comparison processes, differential access to organizational resources, or individualized arrangements with supervisors. As a result, organizational climate perceptions may account for different retirement experiences between and within organizations. In line with these theoretical considerations, we develop hypotheses on how organizational (at the organization level) and relative perceptions of supportive climates (at the individual level) are associated with involuntary retirement.

Organizations differ in how supportive their climates are toward older workers, and supportive climates can increase employees’ agency in shaping their transition from work to retirement (Henkens & Marabini, 2026; Moen, 2013). For example, organizational climates in support of personal development encourage older workers to take up training opportunities, increasing their employability (Li et al., 2023). Similarly, climates in support of health enable workers to tailor job tasks to their needs, reducing daily work obstacles (Gignac et al., 2024; Vanajan et al., 2020). Finally, opportunities for post-retirement work signal employers’ willingness to retain employees longer in the organization.

*Hypothesis 1a (between organizations):* Supportive organizational climates for development, accommodation, and post-retirement work are negatively associated with involuntary retirement, as opposed to voluntary retirement.

Within organizations, employees’ perceptions of available policies and practices may differ. Workplaces are, beyond physical spaces, social contexts. Differences in individual perceptions of organizational climates may stem from social comparison processes (Smith et al., 2012). Older employees often compare themselves to their colleagues in different units, departments, or teams (Bolino & Turnley, 2009). Moreover, some workers can arrange personalized, ad hoc agreements with work supervisors–so-called “I-deals” (Bal & Jansen, 2015; Jonsson et al., 2021). These arrangements may increase perceptions of support among those who secure them, while reducing them among workers unable to access them. Employees may also interpret organizational practices differently based on their own experiences and considerations. Consequently, employees who feel well supported may experience greater freedom to adapt their late careers to personal needs and, as a result, perceive their retirement as voluntary.

*Hypothesis 1b (within organizations):* Relative perceptions of supportive organizational climates for development, accommodation, and post-retirement work are negatively associated with involuntary retirement, as opposed to voluntary retirement.

### Organizational climates and involuntary retirement reasons

The retirement literature suggests that older workers experience retirement as involuntary for different reasons, operating at the micro-, meso-, and macro-levels of analysis. At the micro-level, an illness that prevents older employees from performing work tasks may lead to perceptions of involuntary retirement due to poor health (Szinovacz & Davey, 2005; Van Solinge & Henkens, 2007). Previous research shows that older retirees who retired permanently due to a health condition were eight times more likely to perceive their retirement as involuntary (Denton et al., 2013). At the meso-level, organizational circumstances such as budget cuts and restructuring can result in personnel reductions. One acceptable way to trim personnel is to offer early retirement opportunities to older workers nearing retirement age, resulting in individuals feeling pressured to retire earlier than desired (Clarke, 2007). Such pressures can represent a major constraint on agency in the retirement process. Finally, at the macro level, mandatory retirement regulations limit opportunities to work past the state pension age. Currently, half of the OECD member countries, including the Netherlands, have mandatory retirement practices in place (OECD, 2025b, p. 36). Reaching this age may result in constrained agency for older employees who would rather continue working (Fritz, 2024). This list, while not exhaustive, captures prevailing constraints across individual, organizational, and institutional levels.

Because organizational policies serve different purposes, they likely shape perceptions of involuntary retirement differently depending on the underlying process. Organizational and relative perceptions of supportive development and accommodation climates extend the agency of older employees by increasing their *employability* (Fleischmann et al., 2015). Employers are increasingly aware of the long-term need for age management (Turek et al., 2020). Previous research shows that work organizations characterized by age-friendly organizational climates perform better and retain more employees (Boehm et al., 2014). Development practices, such as employer-sponsored training, prevent skill obsolescence, while accommodation practices, including workload reductions and ergonomic adjustments, help offset age-related functional declines. Investments in older workers’ employability may also improve how retirement transitions are managed. Clear communication around organizing remaining work years may be more effective, reducing conflict and misunderstandings. As a result, involuntary retirements due to health and organizational reasons may occur less frequently in such organizations. Within organizations, employees who perceive greater support for their employability through development and accommodation may have greater opportunities to increase their employability compared to their colleagues. As a result, they may retire less often involuntarily due to poor health and organizational pressures.

*Hypothesis 2a (employability hypothesis, between organizations):* Supportive organizational climates for development and accommodation are negatively associated with involuntary retirement due to health and organizational pressures, as opposed to voluntary retirement.

*Hypothesis 2b (employability hypothesis, within organizations):* Relative perceptions of supportive development and accommodation climates are negatively associated with involuntary retirement due to health or organizational pressures, as opposed to voluntary retirement.

Opportunities for development and accommodation remove obstacles to continued work participation and may increase willingness to remain employed in the organization. However, when employees reach the state pension age, employers have more opportunities to terminate the relationship by enforcing mandatory retirement (OECD, 2025b, p. 36). As a result, in organizations with positive development and accommodation climates, involuntary retirement due to reaching the quasi-mandatory retirement age may occur more frequently. Similarly, employees with more positive perceptions of development and accommodation climates may feel more attached to their jobs and consequently experience retirement at state pension age as involuntary.

*Hypothesis 3a (employability hypothesis, between organizations):* Supportive organizational climates for development and accommodation are positively associated with involuntary retirement due to reaching the quasi-mandatory retirement age, as opposed to voluntary retirement.

*Hypothesis 3b (employability hypothesis, within organizations):* Relative perceptions of supportive development and accommodation climates are positively associated with involuntary retirement due to reaching the quasi-mandatory retirement age, as opposed to voluntary retirement.

Organizational climates for post-retirement work extend older workers’ agency in the retirement transition by increasing their *opportunities for extending working life*. Managers may openly advertise opportunities to work beyond the state pension age, for example, due to personnel shortages or better knowledge transfer. Such possibilities extend the agency of older employees and are expected to prevent involuntary retirement. Employers often negotiate post-retirement work opportunities individually, targeting employees with valuable skills (Bal & Jansen, 2015; Dingemans et al., 2016; Jonsson et al., 2021). Consequently, employees with positive perceptions of these opportunities are less likely to experience retirement at the state pension age as involuntary.

*Hypothesis 4a (extending working life hypothesis, between organizations):* A supportive organizational climate for post-retirement work is negatively associated with involuntary retirement due to reaching the quasi-mandatory retirement age, as opposed to voluntary retirement.

*Hypothesis 4b (extending working life hypothesis, within organizations):* Relative perceptions of supportive organizational climate for post-retirement work are negatively associated with involuntary retirement due to reaching the quasi-mandatory retirement age, as opposed to voluntary retirement.

Given the less straightforward relationship between post-retirement work opportunities and health- or organizational pressure-related retirement, we do not make hypotheses in this regard. Table 1 summarizes hypothesized relationships.

**Table 1:** Hypothesized relationships between organizational climates and involuntary retirement.

Organizational climate type	Overall perceptions of involuntary retirement (H1)	Involuntary retirement due to poor health (H2)	Involuntary retirement due to organizational pressures (H2)	Involuntary retirement due to reaching the quasi-mandatory retirement age (H3/H4)
Development climate	−	−	−	+
Accommodation climate	−	−	−	+
Post-retirement work climate	−	n.d.	n.d.	−


*Note*. “−” = a hypothesized negative relationship; “+” = a hypothesized positive relationship; n.d. = not defined.

## Method

We use data from three waves of the NIDI Pension Panel Survey, a longitudinal, multi-level study conducted among Dutch older workers (Henkens et al., 2017). The study has a stratified design. In a first step, a sample was drawn from the total of organizations affiliated with three large pension funds (ABP, PfZW, BpfBouw) covering half of the Dutch workforce, stratified by size and sector. To obtain sufficient variation in terms of organizational size, it was decided that the organizational sample of each pension fund would include 300 small organizations, 200 medium-sized organizations, and 50 large organizations. In a second step within the medium- and larger-sized selected organizations, a random sample was drawn from birth cohorts 1950–1955 who worked at least 12 hr (Statistics Netherlands, 2015). In the smaller organizations, all employees have been sampled. The three waves of data collection took place in 2015, 2018, and 2023. Questionnaires were sent to 15,480 older workers, and 6,793 were returned in the first wave (44% response rate), 5,312 in the second wave (79%), and 4,258 in the third wave (83%).

For the current study, we excluded respondents who filled in a short version of the questionnaire, excluding some of the organizational climate items (*n *= 498). Moreover, to justify representativeness at the organizational level, we excluded participants in organizations with a response rate lower than 20% and with fewer than three respondents (*n *= 2,170), following the approach used by Li et al. (2023). This leaves a sample of 4,125 older workers nested in 501 organizations who returned the first-wave questionnaire. For participants who retired between Wave 1 and Wave 2 (*n *= 2,292), the predictors were taken from Wave 1, and the dependent variable was taken from Wave 2. For participants who were still working in their career job at Wave 2 and retired between Wave 2 and Wave 3 (*n *= 1,443), the predictors were taken at Wave 2, and the dependent variables were taken at Wave 3. Then, the information was pooled. Due to panel attrition (*n* = 317) and missingness in the dependent variable (*n* = 73), the resulting final sample consisted of 3,735 older workers nested in 501 organizations.

### Measures: dependent variables

#### Involuntary retirement

Retired participants were asked in Waves 2 and 3 of the survey: “Was your decision to stop working voluntary or not?” They could respond: “Yes, it was fully voluntary,” “No, it was partly involuntary,” or “No, it was fully involuntary.” To construct a binary indicator, answers were recoded as “Voluntary” and “Involuntary.”

#### Involuntary retirement reasons

To create an indicator of involuntary retirement reasons, participants who retired involuntarily were asked a follow-up question: “What reason made your decision (partly) involuntary?” In both waves, they could choose “Ill health,” “Pressures from employer,” “Reached the state pension age,” or “Other reasons.” Only in Wave 2 were more reasons defined (“My partner’s health,” “Difficult to combine work and care,” “Pressures from co-workers,” “My partner wanted me to stop”). Pressures from co-workers were included in the pathway consisting of organizational pressures, while the other categories were counted as other reasons due to their low incidence. Respondents could select multiple options. If they reported involuntary retirement due to poor health (*n *= 198), organizational pressures (*n *= 236), or reaching the state pension age (*n *= 280), also in combination with “Other reasons,” they were included as separate categories. If they reported a combination of “Health and organizational reasons” (*n *= 134) or “Organizational reasons and reaching the state pension age” (*n *= 63), they were analyzed as separate outcomes and reported in Supplementary Table. Few respondents selected “Poor health and reaching the state pension age” (*n* = 17) and solely “Other reasons” (*n* = 50); hence, these analyses are not presented. Voluntary retirees (*n* = 2,757) were the baseline category.

### Measures: independent variables

Our main independent variables consist of collective and individual perceptions of organizational climates. Following a referent-shift consensus model (Chan, 1998), we aggregated individual perceptions to create organization-level climate measures. Within-group agreement indexes confirmed that respondents within organizations rated their climates similarly, justifying this aggregation (see Supplementary Table S2 for details). We applied group-mean centering to individual climate scores by subtracting the organizational mean from the individual climate perceptions (Hofmann & Gavin, 1998; Hox et al., 2017, p. 50). This partitions variance into two components: between-organization (reflecting differences in collective organizational climates) and within-organization (reflecting individual perceptions relative to their organizational context). This decomposition allows us to test whether organizational and relative climate perceptions have distinct effects on involuntary retirement. Detailed information is provided in Supplementary Table S3.

#### Control variables

Individual control variables included age, gender, marital status, educational level, supervisory position, presence of chronic conditions, and caregiving burden. Organizational control variables included company size and sector. We also controlled for the presence of a policy that temporarily suspended mandatory retirement regulations for civil servants who retired between 2008 and 2019 (Oude Mulders, 2019).

#### Missing values

All independent variables containing missing information were imputed with multiple imputation by chained equations (20 datasets). For multi-item scales, we imputed at the item level (Eekhout et al., 2014).

Detailed information about all measures is provided in Supplementary Table S3.

### Analysis

To address Hypothesis 1, we estimated a multilevel logistic regression model, in which the binary outcome variable is involuntary versus voluntary retirement. To address Hypotheses 2–4, we adopted a multilevel multinomial regression design to analyze reasons for involuntary retirement, estimating a generalized linear model for multinomial outcomes with random effects at the organization level. The explained variance of the estimated model was manually calculated with McFadden’s pseudo-*R*^2^ formula (Hox et al., 2017, p. 146). All analyses were performed in Stata 18.

## Results

Table 2 presents descriptive statistics, indicating that 26% of retirees reported their retirement was involuntary. The most frequent involuntary retirement reason was reaching the state pension age (7%), followed by organizational pressures (6%) and poor health (5%).

**Table 2:** Descriptive statistics of the study sample.

Variable	Voluntary (*n = *2,575)	Involuntary (*n = *978)	Total (*N *= 3,735)
**Reason for involuntary retirement**			
Voluntary			0.74
Poor health		0.20	0.05
Organizational reasons		0.24	0.06
Reaching the state pension age		0.29	0.07
Health and organizational reasons		0.14	0.04
Organizational reasons and reaching the state pension age		0.06	0.02
Other reasons		0.07	0.02
**Individual perceptions training climate^a^^,^^b^**	3.10 (0.85)	2.89 (0.93)	3.04 (0.87)
Missing	0.02	0.01	0.02
**Individual perceptions accommodation climate^a^^,^^b^**	3.31 (0.75)	3.11 (0.84)	3.26 (0.78)
Missing	0.02	0.02	0.02
**Individual perceptions of post-retirement work climate^a^^,^^b^**	2.78 (1.10)	2.47 (1.09)	2.70 (1.11)
Missing	0.03	0.03	0.03
**Organizational development climate**	3.05 (0.42)	3.00 (0.46)	3.04 (0.43)
**Organizational accommodation climate**	3.27 (0.42)	3.22 (0.44)	3.26 (0.43)
**Organizational post-retirement work climate**	2.72 (0.60)	2.64 (0.58)	2.70 (0.60)
**Age**	63.38 (1.45)	62.89 (1.68)	63.25 (1.53)
**Gender**			
Male	0.54	0.52	0.54
Female	0.46	0.48	0.46
**Educational level**			
Low	0.23	0.23	0.23
Medium	0.25	0.26	0.25
High vocational	0.41	0.39	0.41
High academic	0.11	0.12	0.11
Missing	0.01	0.01	0.01
**Supervisory position (Wave 1)**			
No	0.76	0.76	0.76
Yes	0.24	0.24	0.24
Missing	0.01	0.01	0.01
**Chronic health conditions**			
No conditions	0.38	0.26	0.35
One condition	0.28	0.28	0.28
Two or more conditions	0.34	0.46	0.37
**Partner status**			
Partner working	0.40	0.37	0.40
Partner not working	0.41	0.40	0.40
Single	0.19	0.23	0.20
Missing	0.02	0.01	0.02
**Care burden**			
No weekly care	0.79	0.74	0.78
Low burden	0.17	0.18	0.17
High burden	0.04	0.07	0.05
Missing	0.10	0.08	0.10
**Organizational size**			
Small	0.06	0.07	0.06
Medium	0.46	0.47	0.47
Large	0.48	0.46	0.47
**Organization economic sector**			
Government	0.28	0.27	0.28
Education	0.26	0.24	0.26
Construction	0.17	0.15	0.17
Healthcare	0.13	0.15	0.14
Social work	0.15	0.19	0.16
**Mandatory retirement policy**			
Present	0.96	0.98	0.96
Absent	0.04	0.02	0.04

*Note*. Values report proportion or *M* (*SD*). Variables are pooled across waves: for respondents who retired from their career job before Wave 2, values are drawn from Wave 1; for respondents who retired between Waves 2 and 3, values are drawn from Wave 2. Values are based on data prior to multiple imputation.

a Average individual perceptions of organizational climates were calculated on nonmissing items.

b The variable was group-mean centered in the multivariate analysis.

Table 3, Column A summarizes findings from a multilevel logistic regression model examining the effects of organizational and relative climates for development, accommodation, and post-retirement work on involuntary retirement. A supportive organizational climate for development was negatively associated with involuntary retirement (OR = 0.73, 95% CI = [0.58–0.92]), partially supporting Hypothesis 1a. No associations were found between climates for accommodation and post-retirement work. Turning to the within-part of the model, relative perceptions of accommodation climate (OR = 0.85, 95% CI = [0.75–0.97]) and post-retirement work climate (OR = 0.81, 95% CI = [0.75–0.89]) were negatively associated with involuntary retirement, in partial support of Hypothesis 1b.

**Table 3:** Multilevel regression results for overall and reason-specific involuntary retirement.

Variables	(A) Involuntary retirement (ref. voluntary)		(B) Involuntary retirement reasons (ref. voluntary)						(C) Involuntary retirement due to reaching the quasi-mandatory retirement age (restricted sample; ref. voluntary)	
			Poor health		Organizational pressures		Reached the quasi-mandatory retirement age			
	OR	95% CI	OR	95% CI	OR	95% CI	OR	95% CI	OR	95% CI
**Main variables**										
Individual perceptions of development climate	0.91	[0.81, 1.02]	1.24	[0.98, 1.56]	0.82	[0.67, 1.01]	0.90	[0.74, 1.08]	0.90	[0.73, 1.09]
Organizational climate for development	0.73**	[0.58, 0.92]	0.97	[0.62, 1.52]	0.43***	[0.29, 0.64]	0.71	[0.48, 1.05]	0.76	[0.51, 1.13]
Individual perceptions of accommodation climate	0.85*	[0.75, 0.97]	0.71**	[0.56, 0.92]	0.71**	[0.56, 0.91]	1.18	[0.93, 1.49]	1.22	[0.95, 1.56]
Organizational climate for accommodation	0.92	[0.70, 1.20]	1.15	[0.68, 1.94]	0.97	[0.61, 1.55]	1.29	[0.83, 2.01]	1.25	[0.80, 1.96]
Individual perceptions of post-retirement work climate	0.81***	[0.75, 0.89]	0.79**	[0.66, 0.94]	0.78***	[0.66, 0.92]	0.75***	[0.66, 0.87]	0.72***	[0.62, 0.83]
Organizational climate for post-retirement work	0.93	[0.79, 1.09]	0.97	[0.71, 1.32]	0.88	[0.66, 1.17]	0.90	[0.69, 1.16]	0.84	[0.64, 1.08]
**Individual control variables**										
Age	0.82***	[0.78, 0.87]	0.56***	[0.51, 0.63]	0.64***	[0.58, 0.70]	1.53***	[1.38, 1.70]	1.06	[0.92, 1.22]
Gender (ref. male)	0.89	[0.74, 1.08]	0.65*	[0.44, 0.95]	1.12	[0.79, 1.59]	1.19	[0.87, 1.62]	1.22	[0.88, 1.68]
Education (ref. low)										
Medium	0.96	[0.76, 1.20]	0.98	[0.63, 1.53]	0.82	[0.54, 1.24]	1.24	[0.84, 1.83]	1.21	[0.81, 1.82]
High vocational	1.02	[0.81, 1.29]	0.92	[0.58, 1.48]	0.98	[0.65, 1.48]	1.09	[0.74, 1.60]	1.20	[0.80, 1.78]
High academic	1.47*	[1.09, 2.00]	1.24	[0.65, 2.35]	1.02	[0.57, 1.80]	2.08***	[1.32, 3.28]	2.29***	[1.43, 3.69]
Supervisory position (ref. no)	1.09	[0.90, 1.32]	0.91	[0.62, 1.34]	1.93***	[1.40, 2.67]	0.88	[0.63, 1.23]	0.89	[0.63, 1.27]
Chronic health conditions (ref. none)										
One health condition	1.46***	[1.19, 1.79]	2.66***	[1.58, 4.48]	1.32	[0.93, 1.87]	1.12	[0.82, 1.52]	1.12	[0.81, 1.54]
Two or more health conditions	1.86***	[1.54, 2.25]	5.89***	[3.69, 9.40]	1.10	[0.78, 1.55]	0.94	[0.68, 1.28]	0.88	[0.63, 1.22]
Partner status (ref. working partner)										
Partner, not working	1.17	[0.98, 1.40]	1.15	[0.81, 1.64]	1.29	[0.93, 1.78]	1.01	[0.76, 1.36]	1.01	[0.74, 1.37]
Single	1.43***	[1.15, 1.78]	1.37	[0.88, 2.12]	1.42	[0.95, 2.12]	1.11	[0.77, 1.60]	0.91	[0.62, 1.34]
Care burden (ref. no weekly care)										
Weekly care, low burden	1.12	[0.91, 1.38]	1.08	[0.72, 1.62]	1.01	[0.69, 1.50]	1.09	[0.74, 1.60]	1.10	[0.73, 1.66]
Weekly care, high burden	1.51*	[1.07, 2.13]	1.56	[0.86, 2.81]	1.55	[0.87, 2.75]	0.95	[0.45, 1.99]	1.17	[0.53, 2.58]
**Organizational control variables**										
Organizational size (ref. small)										
Medium	0.86	[0.62, 1.20]	0.54*	[0.30, 0.97]	0.90	[0.51, 1.60]	1.27	[0.72, 2.23]	1.34	[0.75, 2.42]
Large	0.78	[0.55, 1.09]	0.47*	[0.26, 0.86]	0.66	[0.37, 1.19]	1.17	[0.66, 2.08]	1.16	[0.64, 2.11]
Organizational economic sector (ref. government)										
Education	0.78	[0.59, 1.02]	1.36	[0.80, 2.30]	0.71	[0.42, 1.19]	0.48***	[0.32, 0.72]	0.54***	[0.35, 0.82]
Construction	0.79	[0.60, 1.05]	0.84	[0.49, 1.45]	1.20	[0.73, 1.97]	0.32***	[0.19, 0.54]	0.43***	[0.25, 0.73]
Healthcare	1.27	[0.94, 1.72]	2.03*	[1.14, 3.61]	2.15***	[1.26, 3.65]	0.52**	[0.32, 0.86]	0.63	[0.38, 1.03]
Social work	1.23	[0.94, 1.62]	1.13	[0.63, 2.03]	2.18***	[1.37, 3.45]	0.69	[0.46, 1.02]	0.67	[0.44, 1.01]
Absence of mandatory retirement	0.44**	[0.24, 0.80]	1.53	[0.58, 4.04]	1.08	[0.36, 3.26]	0.15***	[0.05, 0.43]	0.21***	[0.07, 0.62]
Constant	12.46	(1.74)	32.46	(3.42)	28.27	(3.15)	−28.92	(3.51)	−12.17	(4.44)
Constant (variance)^a^	0.07	(0.05)	0.06	(0.05)					0.00	(0.00)
McFadden pseudo-*R*^2^	0.06		0.13						0.05	
Observations	3,735		3,735						1,785	

*Note*. OR = odds ratio; 95% CI = 95% confidence interval. Columns 1 and 2 present results from multilevel logistic regression models. Columns 3–8 present results from multinomial multilevel models. Columns 9 and 10 present results from a multilevel logistic regression model restricting the sample to respondents who worked until the state pension age. In all models, the reference category is voluntary retirement. Three additional pathways not shown in this table were included as outcomes: Poor health and organizational pressures, Organizational pressures and Reached the state pension age, and Other reasons.

a The intercept variance was constrained to be equal across equations in the multinomial model.

* *p *< .05.

** *p *< .01.

*** *p *< .005.

Table 3, Column B summarizes findings from a multilevel multinomial regression model examining the main effects of organizational and relative climates for development, accommodation, and post-retirement work on involuntary retirement reasons. Hypotheses 2a and 2b predicted that organizational and relative perceptions of climates for development and accommodation would be negatively associated with involuntary retirement due to poor health and organizational pressures. Employees working in organizations with more supportive development climates were less likely to retire involuntarily due to organizational pressures (OR = 0.43, 95% CI = [0.29–0.64]), partially supporting Hypothesis 2a. However, no other significant associations emerged at the organizational level. Within organizations, employees perceiving more supportive climates toward accommodation relative to their colleagues were less likely to retire involuntarily due to poor health (OR = 0.71, 95% CI = [0.56–0.92]) and due to organizational pressures (OR = 0.71, 95% CI = [0.56–0.91]). Hence, Hypothesis 2b was also partially supported. Moreover, relative perceptions of post-retirement work climate were associated with less involuntary retirement due to poor health (OR = 0.79, 95% CI = [0.66–0.94]) and organizational pressures (OR = 0.78, 95% CI = [0.66–0.92]). Hypotheses 3a and 3b, which predicted that organizational and relative climates for development and accommodation would be positively associated with involuntary retirement due to reaching the quasi-mandatory retirement age, were not supported.

Hypotheses 4a and 4b stated that organizational and relative perceptions of climate for post-retirement work would be negatively associated with involuntary retirement due to reaching the quasi-mandatory retirement age. Hypothesis 4a was not supported at the organizational level. However, relative perceptions of post-retirement work climate reduced involuntary retirement at state pension age (OR = 0.75, 95% CI = [0.66–0.87]), in support of Hypothesis 4b. Because only respondents who work until the quasi-mandatory retirement age are at risk of involuntary retirement for this reason, we additionally estimated a multilevel logistic regression model predicting the likelihood of retiring involuntarily–relative to voluntarily–due to reaching the state pension age using this subsample (Table 3, Column C). The findings from this additional analysis are comparable to our main results.

At the individual level, having one or more chronic health conditions is associated with more involuntary retirement. This result is especially pronounced in the health-related reason (OR_one condition_ = 2.66, 95% CI = [1.58–4.48], OR_two or more conditions_ = 5.89, 95% CI = [3.69–9.40]). Higher-educated workers are more likely to retire involuntarily, driven by involuntary retirements that occur due to the state pension age (OR = 2.08, 95% CI = [1.32–3.28]), while older employees in supervisory positions more often retire involuntarily due to organizational pressures (OR = 1.93, 95% CI = [1.40–2.67]). Older single workers were more likely to retire involuntarily compared to workers with a working partner (OR = 1.43, 95% CI = [1.15–1.78]), and respondents with a high caregiving burden were also more likely to experience involuntary retirement compared to respondents without caregiving responsibilities (OR = 1.50, 95% CI = [1.06–2.11]). At the organizational level, civil servants who retired in the years when mandatory retirement was suspended were less likely to retire involuntarily due to reaching the state pension age (OR = 0.15, 95% CI = [0.05–0.43]).

## Discussion

Retirement is a major life course transition in later adulthood. Maintaining control over this transition has important consequences for post-retirement health, adjustment, and well-being. Yet, involuntary retirement remains widespread. Our findings show that poor health, organizational pressures, and reaching the quasi-mandatory retirement age form separate reasons for involuntary retirement. Supportive organizational climates increase choice in the retirement transition, though analyzing different reasons for involuntary retirement reveals differential effects.

Mandatory retirement at the state pension age emerged as the most common pathway into perceived involuntary retirement, underscoring the constraining role of macro-level institutional frameworks in shaping retirement (Moen, 2013). Although mandatory retirement has been abolished in some countries, such as the United States, it remains prevalent in most OECD countries and is common in the Netherlands (OECD, 2025b, p. 36). Our results reveal a tension between increasing life expectancy and work ability on the one hand and retirement policies that restrict individuals from working beyond the quasi-mandatory retirement age on the other. Notably, perceptions of involuntary retirement due to mandatory retirement were more common among academically educated employees, who usually enjoy favorable working conditions that facilitate prolonged employment (Radl, 2013). We also found that the likelihood of involuntary retirement due to reaching the state pension age was much lower in organizations that suspended mandatory retirement. This suggests that abolishing mandatory retirement effectively eliminates a barrier for workers wishing to extend their careers.

Poor health remains a major reason for perceptions of involuntary retirement. Chronic health conditions emerged as the main driver of the health-induced pathway, reflecting how functional limitations and difficulties in performing daily work tasks can force older employees to exit paid work (Denton et al., 2013). At the organizational level, we did not find support for the hypothesis that supportive organizational climates are associated with less involuntary retirement due to poor health, suggesting that general organizational practices play a limited role in preventing health-related work exits. In contrast, employees with positive perceptions of accommodation climate are less likely to experience involuntary retirement due to poor health. This finding indicates that tailored support at the individual level may help employees remain at work, for example, by decreasing work-related health limitations (Vanajan et al., 2020).

Although early retirement incentives have been increasingly restricted nationally, organizational pressures remain an important and distinct reason for involuntary retirement. In organizations that stimulate older workers to engage in learning and skills development, employees are less likely to feel “pushed out.” This suggests that organizations that invest in older workers are also less inclined to selectively target employees approaching retirement when there is a need for downsizing. Moreover, employees who perceive their organizations as relatively supportive of accommodation measures and post-retirement work opportunities are more likely to perceive individual choice in the retirement transition.

While previous research suggests that involuntary retirement due to employer pressures disproportionately affects individuals in lower socio-economic positions (Ebbinghaus & Radl, 2015), our findings suggest that involuntariness perceptions are not as socially stratified. In fact, we see that managers are more often experiencing involuntary work exit due to organizational pressures. This finding may reflect shifts in workplace dynamics over recent decades, as organizations strive to become less centralized and reduce bureaucracy, often resulting in layoffs in middle-management roles.

Our research sheds light on how organizational and relative climate perceptions affect different reasons for involuntary retirement. While practices that encourage skills development reduce employees’ involuntary retirement across organizations, opportunities for accommodation and post-retirement work seem to benefit specific individuals. This finding suggests that development opportunities may represent a tool to support all older employees, while accommodation and post-retirement work options are more effective when individually negotiated, though this may create inequalities (Dingemans et al., 2016; Wainwright et al., 2019).

Despite its noteworthy strengths, our study also has some limitations. First, our measures of organizational climates consisted of a limited number of items. Second, our youngest respondents were aged 60 years at baseline, while we know that health-related involuntary work exits can occur at earlier ages due to other pathways (i.e., unemployment, disability). As a result, the incidence of health-related involuntary retirement may be underestimated. Finally, our study was carried out in the Netherlands, a country with generous pension schemes, strict mandatory retirement policies, and a tight labor market, which limits generalizability to other country contexts.

Many countries are promoting policies to extend working lives by restricting early exit opportunities and increasing state pension ages. This study shows that even in a labor market context with large labor shortages, perceptions of involuntary retirement are widespread. By looking into involuntary retirement reasons, this article contributes to a deeper understanding of constrained choices in later working lives. We showed that not only individual risk factors but also wider organizational circumstances shape individual agency in work exits. Organizational practices aimed at training and developing older workers may help facilitate longer working lives while supporting individual well-being in the retirement transition.

## Supplementary Material

gbag128_Supplementary_Data

## Data Availability

As original work with the data set has not been completed, the data are not publicly available. However, data, analysis code, and research materials are available from the corresponding author upon reasonable request. The study was not preregistered.

## References

[gbag128-B1] Bal P. M. , Jansen P. G. W. (2015). Idiosyncratic deals for older workers: Increased heterogeneity among older workers enhance the need for I-deals. In Bal P. M., Kooij D. T. A. M., Rousseau D. M. (Eds.), Aging workers and the employee–employer relationship (pp. 129–144). Springer International Publishing. 10.1007/978-3-319-08007-9_8

[gbag128-B2] Beehr T. A. (1986). The process of retirement: A review and recommendations for future investigation. Personnel Psychology, 39, 31–55. 10.1111/j.1744-6570.1986.tb00573.x

[gbag128-B3] Boehm S. A. , Kunze F., Bruch H. (2014). Spotlight on age-diversity climate: The impact of age-inclusive HR practices on firm-level outcomes. Personnel Psychology, 67, 667–704. 10.1111/peps.12047

[gbag128-B4] Bolino M. C. , Turnley W. H. (2009). Relative deprivation among employees in lower-quality leader–member exchange relationships. The Leadership Quarterly, 20, 276–286. 10.1016/j.leaqua.2009.03.001

[gbag128-B5] Caines V. , Ertug G., Bordia P., Schleicher D. J. (2025). Retirement and organizations: Advocating organizational responsibility for retirement in practice and scholarship. Journal of Management, 51, 518–535. 10.1177/01492063241303064

[gbag128-B7] Chan D. (1998). Functional relations among constructs in the same content domain at different levels of analysis: A typology of composition models. Journal of Applied Psychology, 83, 234–246. 10.1037/0021-9010.83.2.234

[gbag128-B8] Clarke M. (2007). Choices and constraints: Individual perceptions of the voluntary redundancy experience. Human Resource Management Journal, 17, 76–93. 10.1111/j.1748-8583.2007.00023.x

[gbag128-B9] Damman M. , Henkens K. (2017). Constrained agency in later working lives: Introduction to the special issue. Work, Aging and Retirement, 3, 225–230. 10.1093/workar/wax015

[gbag128-B10] Denton M. , Plenderleith J., Chowhan J. (2013). Health and disability as determinants for involuntary retirement of people with disabilities. Canadian Journal on Aging = La Revue Canadienne du Vieillissement, 32, 159–172. 10.1017/S0714980813000202 23782974

[gbag128-B11] Dingemans E. , Henkens K. (2014). Involuntary retirement, bridge employment, and satisfaction with life: A longitudinal investigation. Journal of Organizational Behavior, 35, 575–591. 10.1002/job.1914

[gbag128-B12] Dingemans E. , Henkens K., Solinge H. V (2016). Access to bridge employment: Who finds and who does not find work after retirement? The Gerontologist, 56, 630–640. 10.1093/geront/gnu182 26035893

[gbag128-B13] Dorn D. , Sousa-Poza A. (2010). “Voluntary” and “involuntary” early retirement: An international analysis. Applied Economics, 42, 427–438. 10.1080/00036840701663277

[gbag128-B14] Ebbinghaus B. , Radl J. (2015). Pushed out prematurely? Comparing objectively forced exits and subjective assessments of involuntary retirement across Europe. Research in Social Stratification and Mobility, 41, 115–130. 10.1016/j.rssm.2015.04.001

[gbag128-B15] Eekhout I. , de Vet H. C. W., Twisk J. W. R., Brand J. P. L., de Boer M. R., Heymans M. W. (2014). Missing data in a multi-item instrument were best handled by multiple imputation at the item score level. Journal of Clinical Epidemiology, 67, 335–342. 10.1016/j.jclinepi.2013.09.009 24291505

[gbag128-B16] Feldman D. C. , Beehr T. A. (2011). A three-phase model of retirement decision making. The American Psychologist, 66, 193–203. 10.1037/a0022153 21341883

[gbag128-B17] Fleischmann M. , Koster F., Schippers J. (2015). Nothing ventured, nothing gained! How and under which conditions employers provide employability-enhancing practices to their older workers. The International Journal of Human Resource Management, 26, 2908–2925. 10.1080/09585192.2015.1004100

[gbag128-B18] Fritz J. M. (2024). Mandatory retirement and involuntary retirement: Addressing a social justice issue. In Zajda J., Vissing Y. (Eds.), Globalisation, cultural diversity and human rights. (pp. 163–183). Springer Nature Switzerland. 10.1007/978-3-031-55478-0_9

[gbag128-B19] Gignac M. A. M. , Bowring J., Shahidi F. V., Kristman V., Cameron J. I., Jetha A. (2024). Workplace disclosure decisions of older workers wanting to remain employed: A qualitative study of factors considered when contemplating revealing or concealing support needs. Work, Aging and Retirement, 10, 174–187. 10.1093/workar/waac029

[gbag128-B20] Heckhausen J. , Schulz R. (1995). A life-span theory of control. Psychological Review, 102, 284–304. 10.1037/0033-295X.102.2.284 7740091

[gbag128-B21] Henkens K. , Marabini C. (2026). Forge healthy pathways to retirement with employer practices: An extended multilevel model. In Wang M. (Ed.), The Oxford Handbook of Retirement (2nd ed.). Oxford University Press. 10.1093/9780197699584.003.0036

[gbag128-B22] Henkens K. , van Solinge H., Damman M., Dingemans E. (2017). Design and codebook of the NIDI pension panel study (NPPS), first wave. Netherlands Interdisciplinary Demographic Institute.

[gbag128-B23] Hofäcker D. , Schröder H., Li Y., Flynn M. (2016). Trends and determinants of work-retirement transitions under changing institutional conditions: Germany, England and Japan compared. Journal of Social Policy, 45, 39–64. 10.1017/S004727941500046X

[gbag128-B24] Hofmann D. A. , Gavin M. B. (1998). Centering decisions in hierarchical linear models: Implications for research in organizations. Journal of Management, 24, 623–641. 10.1177/014920639802400504

[gbag128-B25] Hox J. , Moerbeek M., Schoot R. v. d. (2017). Multilevel analysis: Techniques and applications (3rd ed.). Routledge. 10.4324/9781315650982

[gbag128-B26] Hyde M. , Dingemans E. (2017). Hidden in plain sight? Does stricter employment protection legislation lead to an increased risk of hidden unemployment in later life? Work, Aging and Retirement, 3, 231–242. 10.1093/workar/wax013

[gbag128-B27] Jonsson R. , Hasselgren C., Dellve L., Seldén D., Larsson D., Stattin M. (2021). Matching the pieces: The presence of idiosyncratic deals and their impact on retirement preferences among older workers. Work, Aging and Retirement, 7, 240–255. 10.1093/workar/waab003

[gbag128-B5926014] Joyce W. F., & , Slocum J. (1982). Climate Discrepancy: Refining the Concepts of Psychological and Organizational Climate. Human Relations, 35, 951–971. 10.1177/001872678203501102

[gbag128-B28] Li Y. , Turek K., Henkens K., Wang M. (2023). Retaining retirement-eligible older workers through training participation: The joint implications of individual growth need and organizational climates. The Journal of Applied Psychology, 108, 954–976. 10.1037/apl0001065 36442028

[gbag128-B29] Lössbroek J. , Lancee B., Lippe T. V. D., Schippers J. (2019). Understanding old-age adaptation policies in Europe: The influence of profit, principles and pressures. Ageing and Society, 39, 924–950. 10.1017/S0144686X17001295

[gbag128-B30] Moen P. (1996). A life course perspective on retirement, gender, and well-being. Journal of Occupational Health Psychology, 1, 131–144. 10.1037/1076-8998.1.2.131 9547042

[gbag128-B31] Moen P. (2013). Constrained choices: The shifting institutional contexts of aging and the life course. In New directions in the sociology of aging. National Academies Press (US). 24404636

[gbag128-B32] OECD. (2025a). OECD Employment Outlook 2025: Can we get through the demographic crunch? OECD Publishing. 10.1787/194a947b-en

[gbag128-B33] OECD. (2025b). Pensions at a glance 2025: OECD and G20 indicators. OECD Publishing. 10.1787/e40274c1-en

[gbag128-B34] Oude Mulders J. (2019). Attitudes about working beyond normal retirement age: The role of mandatory retirement. Journal of Aging & Social Policy, 31, 106–122. 10.1080/08959420.2018.1563473 30614412

[gbag128-B35] Radl J. (2013). Labour market exit and social stratification in Western Europe: The effects of social class and gender on the timing of retirement. European Sociological Review, 29, 654–668. 10.1093/esr/jcs045

[gbag128-B36] Radó M. , Boissonneault M. (2020). Short and long-term change in subjective well-being among voluntary and involuntary retirees. The Journal of the Economics of Ageing, 17, 100178. 10.1016/j.jeoa.2018.11.003

[gbag128-B37] Rhee M.-K. , Mor Barak M. E., Gallo W. T. (2016). Mechanisms of the effect of involuntary retirement on older adults’ self-rated health and mental health. Journal of Gerontological Social Work, 59, 35–55. 10.1080/01634372.2015.1128504 26652660

[gbag128-B38] Schneider B. , Ehrhart M. G., Macey W. H. (2013). Organizational climate and culture. Annual Review of Psychology, 64, 361–388. 10.1146/annurev-psych-113011-143809 22856467

[gbag128-B39] Settersten R. A. (2003). Propositions and controversies in life-course scholarship. In Settersten R. (Ed.), Invitation to the life course: Toward new understandings of later life (pp. 15–48). Routledge.

[gbag128-B40] Smith H. J. , Pettigrew T. F., Pippin G. M., Bialosiewicz S. (2012). Relative deprivation: A theoretical and meta-analytic review. Personality and Social Psychology Review: An Official Journal of the Society for Personality and Social Psychology, 16, 203–232. 10.1177/1088868311430825 22194251

[gbag128-B666] Statistics Netherlands. (2015). *Beroepsbevolking (12-uursgrens)*. https://www.cbs.nl/nl-nl/onze-diensten/methoden/begrippen/beroepsbevolking—12-uursgrens--

[gbag128-B41] Stiemke P. , Hess M. (2022). Determinants of (in-)voluntary retirement: A systematic literature review. Journal of European Social Policy, 32, 348–358. 10.1177/09589287221089465

[gbag128-B43] Szinovacz M. E. (2012). A multilevel perspective for retirement research. In Wang M. (Ed.), The Oxford handbook of retirement. (pp. 152–173). Oxford University Press. 10.1093/oxfordhb/9780199746521.013.0070

[gbag128-B44] Szinovacz M. E. , Davey A. (2005). Predictors of perceptions of involuntary retirement. The Gerontologist, 45, 36–47. 10.1093/geront/45.1.36 15695416

[gbag128-B45] Turek K. , Oude Mulders J., Henkens K. (2020). The proactive shift in managing an older workforce 2009–2017: A latent class analysis of organizational policies. The Gerontologist, 60, 1515–1526. 10.1093/geront/gnaa037 32364231 PMC7681210

[gbag128-B46] Van Dalen H. P. , Henkens K., Wang M. (2015). Recharging or retiring older workers? Uncovering the age-based strategies of European employers. The Gerontologist, 55, 814–824. 10.1093/geront/gnu048 24898558

[gbag128-B47] Van Solinge H. , Henkens K. (2007). Involuntary retirement: The role of restrictive circumstances, timing, and social embeddedness. The Journals of Gerontology. Series B, Psychological Sciences and Social Sciences, 62, S295–S303. 10.1093/geronb/62.5.S295 17906173

[gbag128-B48] Vanajan A. , Bültmann U., Henkens K. (2020). Health-related work limitations among older workers—The role of flexible work arrangements and organizational climate. The Gerontologist, 60, 450–459. 10.1093/geront/gnz073 31150535 PMC7117617

[gbag128-B49] Wainwright D. , Crawford J., Loretto W., Phillipson C., Robinson M., Shepherd S., Vickerstaff S., Weyman A. (2019). Extending working life and the management of change. Is the workplace ready for the ageing worker? Ageing and Society, 39, 2397–2419. 10.1017/S0144686X18000569

[gbag128-B50] Welsh J. , Strazdins L., Charlesworth S., Kulik C. T., D’Este C. (2018). Losing the workers who need employment the most: How health and job quality affect involuntary retirement. Labour & Industry: A Journal of the Social and Economic Relations of Work, 28, 261–278. 10.1080/10301763.2018.1522609

