# Supplementary material for: Involuntary retirement and organizational climates: a multilevel study of older workers
Source: J Gerontol B Psychol Sci Soc Sci. 2026 Jul 1;81(9):gbag128. doi: 10.1093/geronb/gbag128 (PMC13489713; doi:10.1093/geronb/gbag128)
Supplement: Supplementary file 1 [file gbag128_supplementary_data.zip › JGSS-suppl-Marabini-Henkens-van-Solinge.pdf]

The Journals of Gerontology, Series B: Psychological Sciences and Social Sciences

Supplementary Material: Marabini, Henkens, & van Solinge. Involuntary retirement and organizational climates: A multilevel study of older workers.

Supplementary Table 1. Multinomial multilevel model with extended reasons for involuntary retirement (ref. voluntary retirement)

	Poor health		Organizational pressures		Reached the quasi-mandatory retirement age		Poor health and organizational pressures		Organizational pressures and reaching the quasi-mandatory retirement age	
	OR	***95% CI***	***OR***	***95% CI***	***OR***	***95% CI***	***OR***	***95% CI***	***OR***	***95% CI***
Main variables										
Individual perceptions of training climate	1.24	[0.98,1.56]	0.82	[0.67,1.01]	0.90	[0.74,1.08]	0.70**	[0.53,0.91]	0.80	[0.55,1.17]
Organizational climate for development	0.97	[0.62,1.52]	0.43***	[0.29,0.64]	0.71	[0.48,1.05]	0.94	[0.56,1.59]	0.55	[0.26,1.18]
Individual perceptions of accommodation climate	0.71**	[0.56,0.92]	0.71**	[0.56,0.91]	1.18	[0.93,1.49]	0.68*	[0.50,0.91]	1.24	[0.79,1.94]
Organizational climate for accommodation	1.15	[0.68,1.94]	0.97	[0.61,1.55]	1.29	[0.83,2.01]	0.37***	[0.20,0.70]	1.04	[0.44,2.46]
Individual perceptions of post-retirement work climate	0.79**	[0.66,0.94]	0.78***	[0.66,0.92]	0.75***	[0.66,0.87]	0.92	[0.74,1.14]	0.66***	[0.51,0.86]
Organizational climate for post-retirement work	0.97	[0.71,1.32]	0.88	[0.66,1.17]	0.90	[0.69,1.16]	0.94	[0.63,1.40]	1.27	[0.78,2.06]
Individual control variables										
Age	0.56***	[0.51,0.63]	0.64***	[0.58,0.70]	1.53***	[1.38,1.70]	0.49***	[0.43,0.56]	1.72***	[1.38,2.13]
Gender (ref. male)	0.65*	[0.44,0.95]	1.12	[0.79,1.59]	1.19	[0.87,1.62]	0.80	[0.50,1.28]	0.90	[0.48,1.69]
Education (ref. low)										
Medium	0.98	[0.63,1.53]	0.82	[0.54,1.24]	1.24	[0.84,1.83]	1.09	[0.63,1.90]	1.73	[0.76,3.96]
High vocational	0.92	[0.58,1.48]	0.98	[0.65,1.48]	1.09	[0.74,1.60]	1.10	[0.61,1.97]	1.61	[0.71,3.69]
High academic	1.24	[0.65,2.35]	1.02	[0.57,1.80]	2.08***	[1.32,3.28]	0.60	[0.21,1.72]	4.11***	[1.62,10.43]
Supervisory position (ref. No)	0.91	[0.62,1.34]	1.93***	[1.40,2.67]	0.88	[0.63,1.23]	1.27	[0.79,2.04]	0.77	[0.39,1.54]
Chronic health conditions (ref. none)										
One health condition	2.66***	[1.58,4.48]	1.32	[0.93,1.87]	1.12	[0.82,1.52]	3.20***	[1.67,6.14]	0.77	[0.37,1.60]
Two or more health conditions	5.89***	[3.69,9.40]	1.10	[0.78,1.55]	0.94	[0.68,1.28]	6.02***	[3.32,10.94]	1.77	[0.98,3.20]
Partner status (ref. working partner)										
Partner, not working	1.15	[0.81,1.64]	1.29	[0.93,1.78]	1.01	[0.76,1.36]	1.54	[0.99,2.40]	0.75	[0.38,1.47]
Single	1.37	[0.88,2.12]	1.42	[0.95,2.12]	1.11	[0.77,1.60]	2.05**	[1.23,3.41]	2.66***	[1.37,5.16]
Care burden (ref. no weekly care)										
Weekly care, low burden	1.08	[0.72,1.62]	1.01	[0.69,1.50]	1.09	[0.74,1.60]	0.90	[0.54,1.49]	1.94*	[1.02,3.71]
Weekly care, high burden	1.56	[0.86,2.81]	1.55	[0.87,2.75]	0.95	[0.45,1.99]	1.41	[0.70,2.83]	2.59	[0.96,7.01]
Organizational control variables										
Organizational size (ref. small)										

	Poor health		Organizational pressures		Reached the quasi-mandatory retirement age		Poor health and organizational pressures		Organizational pressures and reaching the quasi-mandatory retirement age	
Medium	0.54*	[0.30,0.97]	0.90	[0.51,1.60]	1.27	[0.72,2.23]	0.92	[0.36,2.32]	0.63	[0.24,1.64]
Large	0.47*	[0.26,0.86]	0.66	[0.37,1.19]	1.17	[0.66,2.08]	0.98	[0.39,2.49]	0.65	[0.25,1.70]
Organizational economic sector (ref. government)										
Education	1.36	[0.80,2.30]	0.71	[0.42,1.19]	0.48***	[0.32,0.72]	1.41	[0.68,2.95]	1.10	[0.49,2.47]
Construction	0.84	[0.49,1.45]	1.20	[0.73,1.97]	0.32***	[0.19,0.54]	1.56	[0.74,3.29]	1.37	[0.52,3.58]
Healthcare	2.03*	[1.14,3.61]	2.15***	[1.26,3.65]	0.52**	[0.32,0.86]	2.67*	[1.26,5.69]	1.38	[0.55,3.51]
Social work	1.13	[0.63,2.03]	2.18***	[1.37,3.45]	0.69	[0.46,1.02]	2.83***	[1.41,5.67]	0.93	[0.38,2.26]
Absence of mandatory retirement	1.53	[0.58,4.04]	1.08	[0.36,3.26]	0.15***	[0.05,0.43]	0.00	[0.00,.]	0.15	[0.02,1.34]
Constant[a]	32.46	(3.42)	28.27	(3.14)	-28.92	(3.51)	43.10	(4.27)	-38.08	(7.19)
Constant (variance)[a]	0.06	(0.05)								
McFadden Pseudo-R2	0.13									
Observations	3735.00									

Note: OR = Odds ratio, 95% CI = 95% Confidence Interval. Columns 1–10 present results from multinomial multilevel models. The reference category is voluntary retirement.

[a] The intercept variance was constrained to be equal across equations in the multinomial model.

* *p* <0.05, ** *p* <0.01, *** *p* <0.005.

Supplementary Table 2. Within-group agreement indexes (Wave 1)

Construct	R^*_{wgJ}		A_{wgJ}		ADM (j)		ADMd(j)	
	Mean	*Median*	*Mean*	*Median*	*Mean*	*Median*	*Mean*	*Median*
Organizational climate for development	0.57	0.62	0.65	0.69	0.64	0.67	0.56	0.57
Organizational climate for accommodation	0.60	0.63	0.69	0.70	0.63	0.64	0.60	0.57
Climate for post-retirement work	0.51	0.54	0.60	0.60	0.72	0.73	0.65	0.67

Note. r*wgJ = interrater agreement index for judges (Lindell & Brandt, 1999); awgJ = average interrater agreement index for judges (Brown & Hauenstein, 2005); ADM(j) = average deviation index for judges; ADMd(j) = average median deviation index for judges (Burke et al., 1999). For r*wgJ and awgJ, ., cutoffs of .00 to .30 is considered lack of agreement, .31 to .50 weak agreement, .51 to .70 moderate agreement, .71 to .90 strong agreement, and .91 to 1.00 very strong agreement (LeBreton & Senter, 2008). For AD indexes, the cutoff for substantive agreement is considered at 0.69 (Smith-Crowe et al., 2013).Measures were calculated using the ira package in Stata 18 (Graf-Vlachy, 2017).

References

Brown, R. D., & Hauenstein, N. M. A. (2005). Interrater Agreement Reconsidered: An Alternative to the rwg Indices. Organizational Research Methods, 8(2), 165–184. https://doi.org/10.1177/1094428105275376

Burke, M. J., Finkelstein, L. M., & Dusig, M. S. (1999). On Average Deviation Indices for Estimating Interrater Agreement. Organizational Research Methods, 2(1), 49–68. https://doi.org/10.1177/109442819921004

Graf-Vlachy, L. (2017). IRA: Stata module to calculate rwg and related interrater agreement indices [Computer software]. https://econpapers.repec.org/software/bocbocode/s458422.htm

LeBreton, J. M., & Senter, J. L. (2008). Answers to 20 Questions About Interrater Reliability and Interrater Agreement. Organizational Research Methods, 11(4), 815–852. https://doi.org/10.1177/1094428106296642

Lindell, M. K., & Brandt, C. J. (1999). Assessing interrater agreement on the job relevance of a test: A comparison of CVI, T, rWG(J)}, and rWG(J)} indexes. Journal of Applied Psychology, 84(4), 640–647. https://doi.org/10.1037/0021-9010.84.4.640

Smith-Crowe, K., Burke, M. J., Kouchaki, M., & Signal, S. M. (2013). Assessing Interrater Agreement via the Average Deviation Index Given a Variety of Theoretical and Methodological Problems. Organizational Research Methods, 16(1), 127–151. https://doi.org/10.1177/1094428112465898

Supplementary Table 3. Coding and psychometric properties, and item description of variables in the study sample

Variable	Coding and psychometric properties	Item description/wording
Reason for involuntary retirement<br>Voluntary<br>Poor health<br>Organizational reasons<br>Reaching the state pension age<br>Health and organizational reasons<br>Organizational reasons and reaching the state pension age<br>Other reasons	Categorical variable indicating the type of retirement transition.	Combined measure consisting of two items. The first item refers to the voluntariness of the transition (“Whas your decision to work voluntary or not?”). The responses were “Voluntary”, “Partly involuntary” and “Fully involuntary”. The two latter categories were combined. The second question asked for the reasons for involuntariness (“What made your decision (partly) involuntary”?). The two items were combined to form the dependent variable of the study. The responses were taken from the second or third wave, depending on when participants retired. The categories “Involuntary, due to poor health and organizational pressures”, “Involuntary, due to organizational pressures and reaching the state pension age”, and “Other combinations and reasons” were not reported in the main analysis.
Individual perceptions development climate[ab]	Continuous variable constructed from individual-level items on a 5-item scale (1 = Strongly agree, 5 = Strongly disagree) and reverse coded.	Obtained by averaging two items: “In this organization managers stimulate older workers to keep their knowledge up-to-date” and “Training of older employees has a high priority in this organization” (α =0.78).
Missing		
Individual perceptions accommodation climate[ab]	Continuous variable constructed from individual-level items on a 5-item scale (1 = Strongly agree, 5 = Strongly disagree) and reverse coded.	Obtained by averaging four items: “If work becomes too burdensome it is possible to decrease workload in this organization”, “If work becomes too burdensome working hours can be adjusted in this organization”, “In this organization a lot of attention in paid to health and safety at work”, “Health and wellbeing of employees are important in this organization (α =0.81).
Missing		
Individual perceptions of post-retirement work climate[ab]	Continuous variable constructed from individual-level items on a 5-item scale (1 = Strongly agree, 5 = Strongly disagree) and reverse coded.	“In this organization you can continue working after the state pension age (AOW)”.
Missing		
Organizational development climate	Continuous variable obtained by averaging individual perceptions of developmental climates at the organization level.	See “Individual perceptions of development climate” above.
Organizational accommodation climate	Continuous variable obtained by averaging individual perceptions of accommodation climates at the organization level.	See “Individual perceptions of accommodation climate” above.

Variable	Coding and psychometric properties	Item description/wording
Organizational post-retirement work climate	Continuous variable obtained by averaging individual perceptions of post-retirement climates at the organization level.	See “Individual perceptions of post-retirement work climate” above.
Age	Continuous variable	“In what year were you born?”
Gender<br>Male<br>Female	Categorical variable, 0 = male, 1 = female.	“Are you a man or a woman?”
Educational level<br>Low<br>Medium<br>High vocational<br>High academic<br>Missing	Categorical variable coded 1-4 1 = Low, 2 = Medium, 3 = High vocational, 4 = High academic.	“What is the highest level of education you completed? 1 = Primary school, 2 = Lower vocational education, 3 = Lower general secondary education, 4 = Intermediate vocational education, 5 = Upper general secondary education, 6 = Higher vocational education, 7 = University. Subsequently re-coded into four categories.
Supervisory position (Wave 1)<br>No<br>Yes<br>Missing	Categorical variable 0 = no, 1 = yes.	“Do you have a supervisory position?”
Chronic health conditions<br>No conditions<br>One condition<br>Two or more conditions	Categorical variable 0 = no health conditions, 1 = one health condition, 2 = two or more health conditions.	Do you have one or more of the following longstanding diseases, conditions, or handicaps (diagnosed by a doctor)? Complaints of the hand or arms, Complaints of the hips, legs, or feet (1/0), Complaints of the back and neck (1/0), Migraine or severe headache (1/0), Cardiovascular diseases (1/0), Ashtma, bronchitis, emphysema (1/0), Gastrointestinal disorders (1/0), Diabetes (0/1), Psychological complaints/disorders (1/0), Life-threatening diseases (i.e., cancer, AIDS) (1/0), Sleep problems (1/0). If respondents ticked one of these conditions they were classified as “One health condition”; if they ticked two ore more “Two or more health conditions”; if they were all 0 or missing they were classified as “No health condition”.
Partner status<br>Partner working<br>Partner not working<br>Single<br>Missing	Categorical variable, 1 = “Partner working” (reference category), 2 = “Partner not working”, 3 = “Single”	Do you have a partner? 1 = “Yes, I am married”; 2 = “Yes, I cohabit with a partner”; 3 = “Yes, I do have a partner but we do not live together”, 4 = “No, I am single”. The answers were recoded in 2 categories, partnered and single. Partnered respondents were asked “Which of the following situations applies to your wife/husband/partner?”. 1 = “Works as employee”, 2 = “Self-employed”, 3 = “Is (early) retired, but still engaged in paid work”, 4 = “Is (early) retired, and not engaged in paid work”, “5 = “Unemployed”, 6 = “Disabled”, 7 = “House husband/wife”. Categories 1, 2, 3 were classified as working partner, and else as not working.

Variable	Coding and psychometric properties	Item description/wording
Care burden<br>No weekly care<br>Low burden<br>High burden<br>Missing	Categorical variable. 1 = “No weekly caregiving” (reference category), 2 = “Weekly caregiving, low burden”, 3 = “Weekly caregiving, high burden”.	Respondents were asked how frequently they provide help to family members or friends who are ill or in need of help. If they provide help, but answered “About monthly” or “About couple of times a year”, they were classified as “No weekly caregiving”. If they answered “Daily or several times a week”, they were considered as weekly caregivers. They were further asked “To what extent is providing that help (care) burdensome?”. Answers 1 = “Very” and 2 = “Fairly” combined with weekly caregiving were classified as “Weekly caregiving, high burden”, and answers 3 = “A little” and 4 = “Not at all” as “Weekly caregiving, low burden”.
Organizational size<br>Small<br>Medium<br>Large	Categorical variable. 1 Small, 2 = Medium, 3 = Large.	Information obtained from the pension funds. Small = less than 50 employees, Medium = between 50 and 250 employees, Large = more than 250 employees.
Organization economic sector<br>Government<br>Education<br>Construction<br>Healthcare<br>Social work	Categorical variable. Coded = 1 Government, 2 = Education, 3 = Construction, 4 = Healthcare, 5 = Social work.	Information obtained from the pension funds.
Mandatory retirement policy<br>Present<br>Absent	Categorical variable 0 = presence of mandatory retirement policy, 1 = absence.	This variable identifies a subgroup of workers for which mandatory retirement policy was abolished from 2008 (national level civil servants).

Note: Variables are pooled across waves: for respondents who retired from their career job before Wave 2, values are drawn from Wave 1; for respondents who retired between Wave 2 and 3, values are drawn from Wave 2. Values are based on data prior to multiple imputation.

[a] Average individual perceptions of organizational climates were calculated on non-missing items.

[b] The variable was group-mean centered in the multivariate analysis.
